# Editorial: The role of immune cells in the progression of autoimmune diseases affecting the CNS

**DOI:** 10.3389/fimmu.2022.1078396

**Published:** 2022-11-09

**Authors:** Czeslawa Kowal, Jelka Pohar, Flora Zavala

**Affiliations:** ^1^ Institute of Molecular Medicine, Feinstein Institutes for Medical Research, Manhasset, NY, United States; ^2^ Molecular Medicine at Zucker School of Medicine at Hofstra/Northwell, Hempstead, NY, United States; ^3^ Immunology and Cellular Immunotherapy, Department of Genetic Toxicology and Cancer Biology, National Institute of Biology, Ljubljana, Slovenia; ^4^ Université Paris Cité, INSERM UMR-S1151, CNRS UMR-S8253, Institut Necker Enfants Malades, Paris, France

**Keywords:** CNS pathophysiology, dendritic cells, mucosal-associated invariant T cells, STAT3, Th17 cells, PP2Cδ, supramolecular organizing centers (SMOCs), neutrophil extracellular traps

Insights into the dynamics of immune responses in immune-privileged tissues such as the central nervous system (CNS) are critical to understanding the etiology of autoimmune diseases. Essential in this field is understanding the ways immune cells access and traffic across different types of the blood-brain barrier (BBB), and how the therapeutics alter these processes (see excellent review by Mapunda et al.). The current Research Topic brought into attention a diverse panel of original research papers and two reviews on the immune cells involved in CNS pathophysiology, including dendritic cells (DC), mucosal-associated invariant T (MAIT) cells, neutrophils, and pathogenic Th17 cells, as well as a different aspects of that pathophysiology, including molecular signaling pathways (PP2Cδ) and the role of signaling complexes, known as supramolecular organizing centers (SMOCs), therapeutic interventions (STAT3-specific nanobody, treatment of rheumatoid meningitis with intravenous immunoglobulin - IVIg), possible new forms of autoantigens (neutrophil extracellular traps (NETs) in neuropsychiatric systemic lupus erythematosus - NPSLE), and assessment of correlation between inflammatory markers and severity of autoimmune encephalitis (AE).

It is becoming increasingly clear that metabolism is intrinsically linked to the function of immune cells and their interaction with other cells (1). DCs represent major contributors to the immune response by presenting antigen and secreting diverse cytokines. Lv et al. demonstrate that ablation of the phosphatase PP2Cδ in DCs unleashed their activation leading to abnormal Th1/Th17 differentiation and exacerbated EAE pathology. PP2Cδ loss led to de-repression of the methyltransferase NSD2, thereby boosting the Rictor/mTORC2 pathway inducing major metabolic changes in DCs such as elevated mitochondrial oxidative phosphorylation and aerobic glycolysis. By inducing ATP-citrate lyase (ACLY), it also stimulated histone acetylation, facilitating the expression of genes characterizing hyperactivated DCs. PP2Cδ therefore exerts a key role in DC activation and function *via* the Rictor/mTORC2/ACLY pathway that controls immune homeostasis *via* metabolic and epigenetic regulations.

MAIT cells are highly conserved unconventional T cells activated by riboflavin-derived bacterial and yeast antigens presented by MHC-related protein 1 (MR1). MAIT cells are of explicit interest in multiple sclerosis (MS) (2), a T-cell-driven, autoimmune inflammatory neurological disease, due to their abundance, intrinsic effector-memory phenotype and capability to produce proinflammatory cytokines. Gargano et al. report an evidence of gut dysbiosis of MS patients compared with healthy donors (HD) by metagenomics and metataxonomic analysis of cultivable faecal samples collected from both groups. MAIT cells derived from MS patients show higher level of activation and proliferation in response to fungal isolates overrepresented in MS group (*C. albicans* and *S. cerevisiae*), and an increased proinflammatory cytokine production by activated innate immune cells. Importantly, MAIT cells containing pro-inflammatory cytokines were detected in the active brain lesions of MS patients.

Low density neutrophils (LDNs) were correlated with disease activity in systemic lupus erythematosus (SLE). Their contribution to pathology in SLE is attributed to their capacity to produce type I IFNs and to spontaneously form NETs containing autoantigens and proinflammatory proteins. In the review Sim et al. assess the role of LDNs in NPSLE; their proinflammatory function and the participation in the opening of the BBB, hence allowing the anti-neuronal pathogenic antibodies entering the brain parenchyma. The pathogenicity of these antibodies in NPSLE and the role of matrix metalloproteinase-9 and neutrophil gelatinase-associated lipocalin in BBB disruption are also evaluated. Finally, NETs as a source of autoantigens, especially oxidized mitochondrial DNA, enabling to sustain or to initiate autoimmune response, as well as possible therapeutic interventions are discussed.

Infiltration of pathogenic T-cells is a hallmark of several autoimmune diseases including uveitis and multiple sclerosis. Mbanefo et al. present a transcription factor STAT3 that plays an important role in the biology of Th1 and particularly Th17 T cells. Although STAT3 is an intracellular protein that is not easily targeted, the authors used the camelid nanobody. In the animal model of experimental autoimmune uveitis (EAU), which targets retina – another immune-privileged tissue, the STAT3-specific antibody was able to efficiently penetrate the blood retina barrier and suppress the differentiation, proliferation, and cytokine release of Th17 cells, leading to EAU amelioration. Authors provide a perspective for the development of future therapeutics to specifically inhibit pathogenic cells, particularly in immune-privileged organs such as the CNS.

Rheumatoid meningitis represents a rare complication of rheumatoid arthritis in the CNS. Zhang et al. report on a case diagnosed on the basis of rheumatoid factor, anti-cyclic citrullinated peptide antibody and IL-6 positivity in the CSF and the serum together with the unexpected presence of anti-NMDAR Abs in the CSF. Brain MRI confirmed hyperintensity of frontal and parietal lobes. IVIg was effective to alleviate clinical symptoms and lesions and restore normal levels of biochemical indicators except for persisting anti-NMDAR Abs.

Autoimmune encephalitis is another inflammatory disease that targets cells of the CNS and causes complex clinical manifestations such as motor dysfunction and psychiatric disorders. Different scoring systems such as the modified Rankin Scale (mRS) or the Clinical Assessment Scale for Autoimmune Encephalitis (CASE) are combined to accurately assess the severity of the disease (3). In addition, inflammatory markers such as neutrophil-to-lymphocyte ratio (NLR) and monocyte-to-lymphocyte ratio (MLR) are becoming increasingly popular to help clinicians monitor disease progression, prognosis, and response to treatment. Liu et al. for the first time demonstrated the positive association between MLR and the severity of AE.

The increasing incidence of neurodegenerative diseases such as Alzheimer’s is a growing public health problem and a burden on society. Misfolded proteins and aggregates cause direct neurotoxicity and contribute to the production of inflammatory mediators *via* activation of pattern recognition receptors, leading to neuroinflammation and, in later stages, BBB damage and infiltration of peripheral immune cells (Chee and Solito). This neuroinflammation is propagated through SMOCs, comprised of ligands, sensors, adaptors, and effectors that form multiprotein complexes such as myddosomes, inflammasomes, and necrosomes. Sušjan-Leite et al. provide a comprehensive overview of SMOCs involved in neuroinflammation and their role in the development of neurodegenerative and neuroinflammatory diseases by linking innate and adoptive immunity.

In conclusion, we hope this Research Topic will stimulate new studies testing and expanding therapeutic potentials reported here, dissecting molecular pathways even further to spare immune protection and to provide base for new clinical trials.

## Author contributions

All authors listed have made a substantial, direct, and intellectual contribution to the work and approved it for publication.

## Funding

This work was supported by the NIH grant 1P01 AI073693-06, Research Fund of The National Institute of Biology (10ICIGEN) and Slovenian Research Agency (P1-0245, J3-3084).

## Acknowledgments

We would like to thank all authors for their contributions to this Research Topic. We also thank all the reviewers for their comprehensive and timely evaluation of the submitted manuscripts.

## Conflict of interest

The authors declare that the research was conducted in the absence of any commercial or financial relationships that could be construed as a potential conflict of interest.

## Publisher’s note

All claims expressed in this article are solely those of the authors and do not necessarily represent those of their affiliated organizations, or those of the publisher, the editors and the reviewers. Any product that may be evaluated in this article, or claim that may be made by its manufacturer, is not guaranteed or endorsed by the publisher.
